# Results of the 2020 Genomic Proficiency Test for the network of European Union Reference Laboratory for Antimicrobial Resistance assessing whole-genome-sequencing capacities

**DOI:** 10.1099/mgen.0.001076

**Published:** 2023-08-01

**Authors:** Thea Kristensen, Lauge Holm Sørensen, Susanne Karlsmose Pedersen, Jacob Dyring Jensen, Hanne Mordhorst, Niamh Lacy-Roberts, Oksana Lukjancenko, Yan Luo, Maria Hoffmann, Rene S. Hendriksen

**Affiliations:** ^1^​ National Food Institute, Research Group of Genomic Epidemiology, Technical University of Denmark, Kgs. Lyngby, Denmark; ^2^​ Department of Plant and Environmental Sciences, Section for Organismal Biology, Faculty of Science, University of Copenhagen, Copenhagen, Denmark; ^3^​ National Food Institute, Research Group of Global Capacity Building, Technical University of Denmark, Kgs. Lyngby, Denmark; ^4^​ Clinical Microbiomics, Copenhagen, Denmark; ^5^​ Center for Food and Safety and Applied Nutrition, US Food and Drug Administration, College Park, Maryland, USA

**Keywords:** Statistical comparison, Short-read sequencing, Genomic Proficiency Test, Whole Genome Sequencing, Quality control, Pathogenic bacteria

## Abstract

The global surveillance and outbreak investigation of antimicrobial resistance (AMR) is amidst a paradigm shift from traditional biology to bioinformatics. This is due to developments in whole-genome-sequencing (WGS) technologies, bioinformatics tools, and reduced costs. The increased use of WGS is accompanied by challenges such as standardization, quality control (QC), and data sharing. Thus, there is global need for inter-laboratory WGS proficiency test (PT) schemes to evaluate laboratories' capacity to produce reliable genomic data. Here, we present the results of the first iteration of the Genomic PT (GPT) organized by the Global Capacity Building Group at the Technical University of Denmark in 2020. Participating laboratories sequenced two isolates and corresponding DNA of *Salmonella enterica *, *Escherichia coli* and *Campylobacter coli *, using WGS methodologies routinely employed at their laboratories. The participants' ability to obtain consistently good-quality WGS data was assessed based on several QC WGS metrics. A total of 21 laboratories from 21 European countries submitted WGS and meta-data. Most delivered high-quality sequence data with only two laboratories identified as overall underperforming. The QC metrics, N50 and number of contigs, were identified as good indicators for high-sequencing quality. We propose QC thresholds for N50 greater than 20 000 and 25 000 for *Campylobacter coli* and *Escherichia coli,* respectively, and number of contigs >200 bp greater than 225, 265 and 100 for *Salmonella enterica *, *Escherichia coli* and *Campylobacter coli *, respectively. The GPT2020 results confirm the importance of systematic QC procedures, ensuring the submission of reliable WGS data for surveillance and outbreak investigation to meet the requirements of the paradigm shift in methodology.

## Data Summary

Sequence data from this project have been submitted to ENA under project accession number PRJEB58706, samples can be found under accession numbers ERS14601265-ERS14601457. Closed reference genomes are available under accession numbers found in Table 1. Obtained metric statistics and single nucleotide polymorphism (SNP) counts are available in the Supplementary Material and the full method is described in detail in the Supplementary Methods file, available in the online version of this article.

**Impact Statement**

With this article we provide the first glimpse into the workflow of European reference laboratories for applying WGS in AMR surveillance. It gives insights on the methods used for obtaining WGS and the quality of the obtained data. In addition, we analyse several QC metrics and present specific thresholds for the metrics N50 and number of contigs, commonly used to ensure high-quality data in WGS. The study covers two isolates of *Salmonella enterica *, *Escherichia coli* and one of *Campylobacter coli *, but the methods used are generic and can be considered as descriptive of WGS for AMR in general. With the application of WGS gaining ever more ground for AMR surveillance, it is of upmost importance that workflows are standardized and perfected to minimize the probability of error. Initiatives such as the GPT (presented here) are necessary to illuminate practices in need of improvement. Not only as a one-time evaluation, but at an ongoing basis to continuously improve methods and ensure the quality of obtained data.

## Introduction

In 1995, the first two complete bacterial genomes, *Haemophilus influenzae* and *Mycoplasma genetalium* were published [1, 2] and whole-genome sequencing (WGS) has transformed the landscape of microbiology ever since. Recent developments in sequencing technologies and bioinformatics tools combined with the steady decline of sequencing costs have made WGS an advanced yet viable solution for epidemiologic investigation and surveillance of bacterial pathogens [3–5].

Several WGS platforms have been developed with different technical specifications and costs (World Health Organization [6]), marketed towards either addressing sequencing yield (through-put) or providing longer reads. Similarly, numerous bioinformatics pipelines and tools have been established for data handling and analysis, of which some are web-based and/or command line and open source, as opposed to graphical user interface and commercial programmes, allowing for inter-laboratory standardization. These include timely inference of bacterial subtyping and antimicrobial resistance (AMR) profiling, traditionally determined by time-consuming laboratory procedures. Today, these technologies are employed in different combinations by individual researchers and clinical bioinformaticians around the world [7].

The increased use of WGS conveys several challenges such as standardization, quality control (QC), data sharing, curation of databases, and analysis. To continuously monitor the extent of these challenges, there is a need for an inter-laboratory WGS proficiency test (PT) scheme to meet the demand in evaluating laboratories' capacity to produce reliable genomic data [8]. This has been addressed by the Global Microbial Identifier (GMI) initiative [9], a global network of scientists and other experts committed to improving global infectious disease and food safety prevention using WGS (https://www.globalmicrobialidentifier.org/). Since the GMI initiative was established in 2011, several proficiency tests have since been executed with the main objective of facilitating the production of reliable WGS results of consistently good quality [10]. Here, we present and evaluate the results of the first iteration of the Genomic Proficiency Test (GPT2020) organized by the Research Group for Global Capacity Building at the Technical University of Denmark, National Food Institute (DTU Food) in 2020. The participating laboratories ability to obtain consistently good-quality WGS data was assessed based on several QC WGS metrics, single nucleotide polymorphism (SNP) analysis and multilocus sequence typing (MLST) prediction. Another goal of the GPT2020 was to set QC thresholds for several parameters, which will serve as quality guidance in future self-evaluation of WGS data by laboratories.

## Methods

### Organization

The GPT2020 was coordinated by DTU Food in its capacity as the European Union Reference Laboratory for Antimicrobial Resistance (https://www.eurl-ar.eu/).

### Reference strains

Two isolates of three bacterial species with resistance mechanisms of interest to the EURL-AR network were selected: *Salmonella enterica (S. enterica*), *Escherichia coli (E. coli*) and *Campylobacter coli (C. coli*). An overview of the strains and their details are found in Table 1.

**Table 1.** Strain overview

ID	Species	Sequence type	Total basepairs	Contigs	N50	GC%	Accession numbers	Source
GPT2020-001	* S. enterica *	13	5 166 962	4	4 790 899	51.70	OX442403.1- OX442406.1	This study
GPT2020-002	* S. enterica *	469	5 014 670	1	5 014 670	52.05	OX442412.1	This study
GPT2020-003	* E. coli *	4980	5 223 867	5	4 899 319	50.42	GCA_016925115	PRJNA230969
GPT2020-004	*E.coli*	2179	5 187 126	4	4 887 198	50.70	OX442407.1- OX442410.1	This study
GPT2020-005	* C. coli *	1117	1 805 725	2	1 766 438	31.20	OX442413.1- OX442414.1	This study
GPT2020-006	* C. coli *	3336	1 814 825	1	1 814 825	31.31	OX442411.1	This study

Overview of strains used in the DTU Genomic Proficiency Test 2020, showing the identified species, sequence type (ST), total expected genome size, contigs (note this is the number of plasmids plus chromosome), N50, GC content and accession number on the European Nucleotide Archive (ENA).

Genomic DNA (gDNA) was extracted from the six GPT2020 isolates using an Invitrogen Easy-DNA KitTM (Invitrogen, Carlsbad, CA, USA). The gDNA of each strain was dried and supplemented with DNA stabilizing agent (DNAstable Plus, Biomatrica, https://www.biomatrica.com/download/hp-dnastable-plus-handbook/).

The GPT2020 strains were sequenced using the Sequel system (Pacific Bioscience, CA, USA) to obtain a closed reference genome and analysed using the bioinformatics tools to confirm the MLST and sequence type (ST) genes using the pipeline MLST v 2.0.4 [11] available from Center for Genomic Epidemiology http://www.genomicepidemiology.org/

### Distribution of reference material

The reference material; two isolates of each of the three species (GPT2020-001 BACT to GPT2020-006 BACT) and corresponding gDNA (GPT2020-001 DNA to GPT2020-006 DNA) was distributed by the EURL-AR to each participating laboratory on 22 September 2020.

### Procedure

Participants were asked to perform the GPT2020 using the WGS methodologies routinely employed at their laboratories and answer questions about the methodology applied via an online survey (Table S1).

### Sequencing quality analysis

Participants were requested to submit their generated WGS raw sequence files in fastq format to a DTU Food hosted ftp-site. The raw reads have been submitted by the GPT2020 provider to the European Nucleotide Archive (ENA) under project accession number PRJEB58706.

The reads of the GPT2020 were run through an in-house pipeline, trimmed using BBDuk2 (part of BBmap tools v36.49, https://sourceforge.net/projects/bbmap/), *de novo* assembled using SPAdes v 3.9.0 software [12] and aligned to the reference genome using the Burrows-Wheeler Aligner (BWA)-MEM algorithm v 0.7.12 [13] with default settings. Samtools v 1.2 [14] was used to filter the reads that did not map. Finally, each of the QC parameters listed in Table 2 were calculated. The statistical analysis of each calculated QC metric was visualized in 12 boxplots (Fig. S1).

**Table 2.** QC metrics

QC metric	Adjusted quality threshold
Average insert size	Less than 3SD levels below mean
Numbers of reads (for paired-end reads, the total numbers of reads is calculated as the sum of reads in the two files)	
Numbers of reads after trimming (for paired-end reads, the total numbers of reads is calculated as the sum of reads in the two files)	
Number of reads that map to the total reference DNA (chromosome +any plasmids) using BWA	
Number of reads that map to reference chromosome	
Number of reads that map to plasmids, if any	
Numbers of unmapped reads	
Proportion (%) of reads that map to total reference DNA	Less than 3SD below mean
Coverage of the reference chromosome (fraction of chromosome positions that were covered by at least one read pair).	Less than 3SD below mean
Depth of coverage of total reference DNA	
Depth of coverage of the reference chromosome	
For the assemblies, the following QC parameters were calculated:	
Total size of assembly (bp) (all contigs)	
Proportion (%) of size of assembly that map to the total size of the reference DNA	More or less than 3SD levels from mean
Total number of contigs	
Number of contigs with a length above 200 bp	More than 3SD levels above mean
N50 (defined as the length of the shortest contig, in the set of largest contigs that represents at least 50 % of the assembly)	Less than 3SD levels below mean
NG50 (defined as the length of the shortest contig, in the set of largest contigs that represents at least 50 % of the reference genome)	Less than 3SD levels below mean
Q-score for forward and reverse reads separately	Less than 3SD levels below mean
MLST	Inability to determine sequence type
SNP count	More than 10 SNPs

Overview of QC metrics calculated in the GPT20 and their adjusted quality thresholds.

The MLST of each isolate was predicted using a command line version of CGE MLST v 2.0.4 [11]. SNPs were determined and filtered for exclusion using the CGE pipeline CSI phylogeny v.1.4 available at (https://cge.cbs.dtu.dk/services/CSIPhylogeny/) as described by [15]. Possible contamination was investigated with Kraken2 on raw sequence data [16].

### Correlation statistics

Samples larger than 105 % or smaller than 95 % of assembly size compared to the closed reference genomes or with incorrect or incomplete MLST-scheme genes identified were removed (Table 3).

**Table 3.** Laboratory performance

	Percentage of coverage of the reference chromosome	Percentage of reads mapped to the reference genomes.	Samples removed due to genome size >105 %	Samples removed due to coverage of chromosome<95 %	Average insert size	Percentage in size compared to the reference genomes	Phred quality score (*Q* score forward and reverse)	No. of contigs >200 bp	N50/NG50	Assigned MLST	SNP difference greater than 10 SNPs
User_1											
User_2			X			X		X			
User_3											X
User_4											
User_5	X					X		X			
User_6			X								X
User_7											
User_8	X										
User_9	X	X		X						X	
User_10		X	X		X		X		X		X
User_11											
User_12			X								
User_13											
User_14	X			X						X	
User_15											
User_16			X							X	
User_17											X
User_18											
User_19			X								
User_20	X										X
User_21											

Overview of laboratory underperformance across selected QC metrics and exclusion criteria. X marks laboratories with results identified as outliers or below the adjusted quality threshold. Correlated metrics are highlighted in matching colours, blue indicates group 1 correlated metrics, green indicates group 2 correlated metrics and laboratories highlighted in orange were identified as general underperformers.

**Fig. 1.**
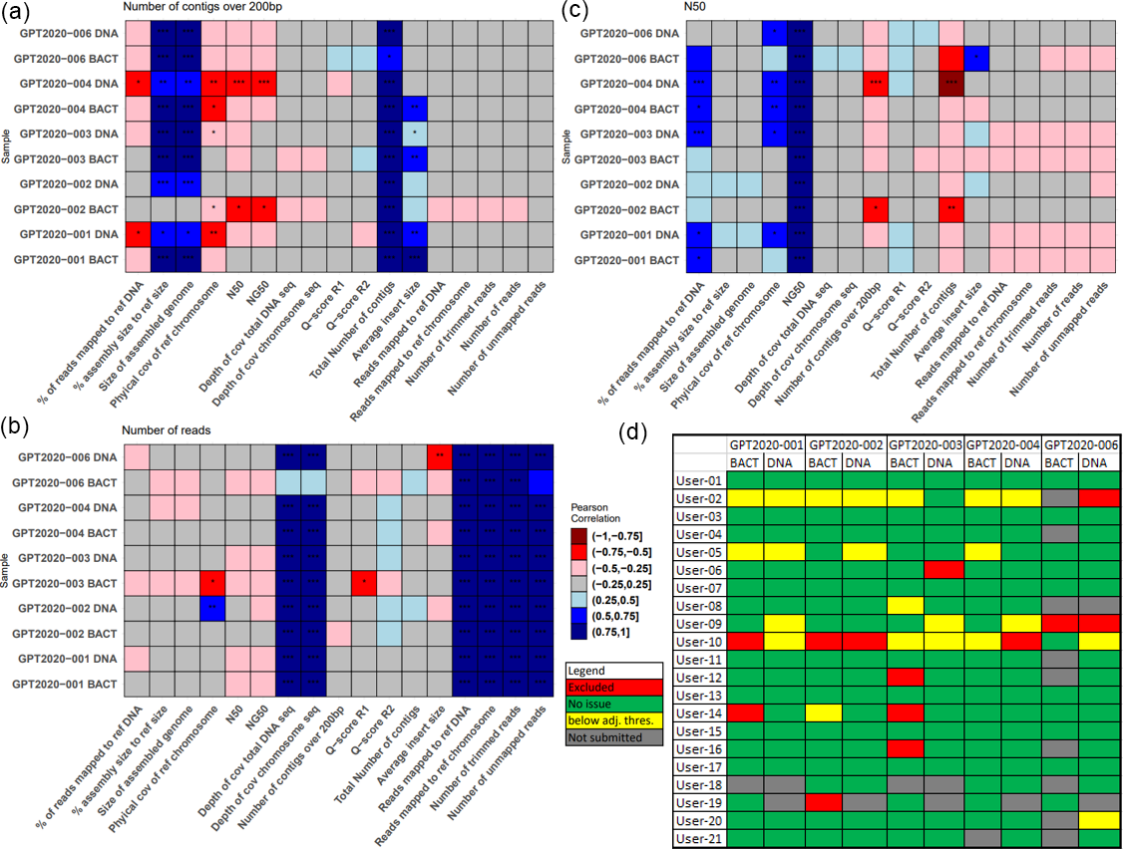
Correlation and genome submissions. Heatplots for correlations between (a) number of contigs over 200 bp, (**b**) number of reads and (c) N50 and all other calculated parameters for each type of genome. Significance is indicated by stars, where one, two and three stars corresponds with <0.05, <0.01 and <0.001, respectively. Blue indicates positive and red negative Pearson correlation. (**d**) provides an overview of submitted genomes by each participant, with colours indicating the following: red=preliminary exclusion, yellow=failed adjusted quality threshold, green=no issues found, grey=not submitted.

Isolates that deviated by more than 3SD from the mean for each sample type were then removed. Linear regression analysis was then performed on all QC parameters found in Table 2, using the linear model (lm) in R. Accepted correlation had a *P*-value less than 0.05 and Pearson correlations were calculated using the in-built R function (cor). The correlations were visualized using the R-libraries reshape2 and ggplot2 (Fig. 1a–c).

**Fig. 2.**
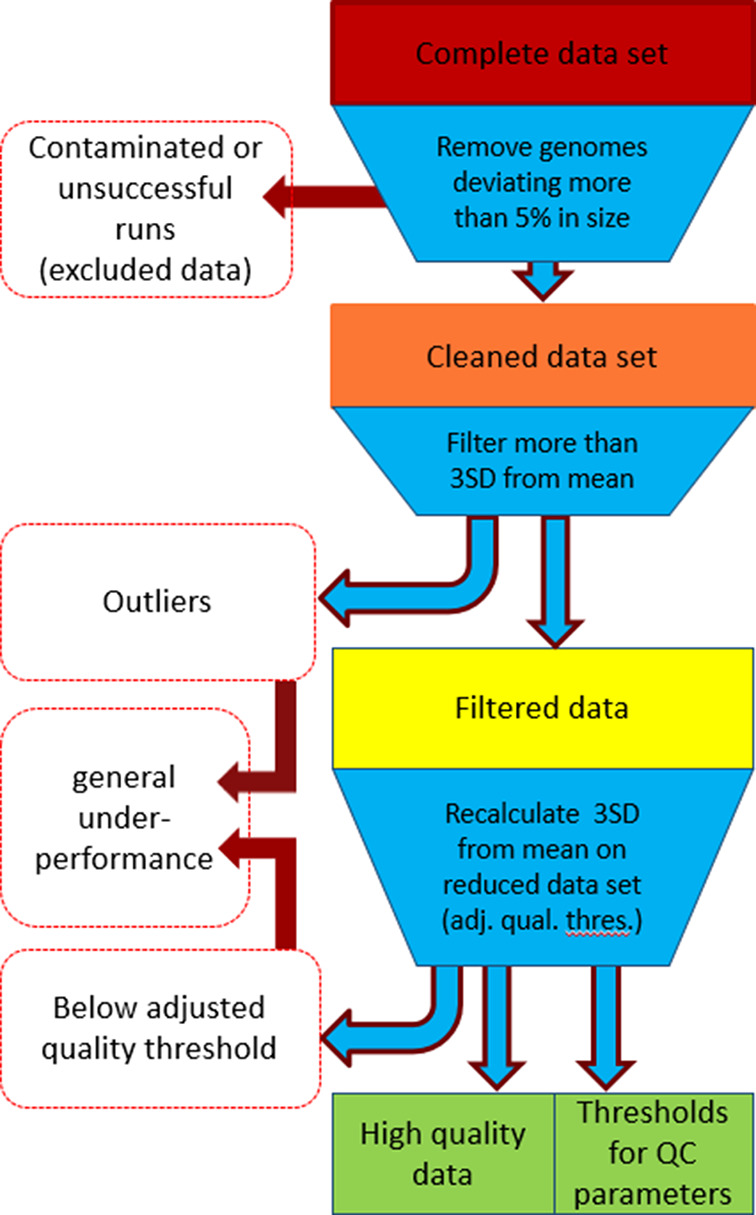
Data filtration workflow. Data was filtered in three steps. (1) The data was cleaned based on genome size and MLST. (2) Outliers were identified in the dataset based on 3SD from the mean. (3) 3SD was recalculated discounting previously identified outliers, termed adjusted quality threshold. Participants below the adjusted quality threshold where flagged. Flags across uncorrelated QC metrics was termed ‘general laboratory underperformance’.

The SD and mean were recalculated for the cleaned dataset. A threshold of 3SD below/above the mean, depending on the specific QC-metric (referred to as adjusted quality threshold below) was set to define minimum QC-thresholds for acceptable sequence quality (see Table 2 and Fig. 2).

Difference in means between live cultures and purified DNA samples were tested for significance (*P*-value less than 0.05 *P*-value). Distributions of number of contigs >200 bp were compared using Welch’s *t*-test (t.test) and N50s were tested using Kolmogorov–Smirnov test (ks.test) in R.

### General underperformance

To identify laboratories that underperformed overall, a subset of the QC metrics was selected based on the correlation statistics. Failure across uncorrelated metrics was defined as general laboratory underperformance.

For more details, see File S1.

## Results

### Participation

A total of 21 national reference laboratories (NRLs) submitted sequence and meta-data, however, not all participants tested all the strains provided. Fig. S2 visualizes which countries provided data for at least one of the PT components.

### Initial evaluation

The submission of raw reads from User-18’s GPT2020-001 (BACT) and −003 (DNA), were excluded from the statistical analysis when initially evaluated due to insufficient assembly (data not shown). A total of 223 genome submissions were considered for further statistical analysis.

### Methodologies and technologies used by participants

The storage temperature [−20 °C, 4 °C, no storage time (processed immediately upon receipt), room temperature] of the bacterial cultures (BACT) and DNA differed between the laboratories. Participants mostly stored the bacterial cultures either at 4 °C (*n*=12, 57%) or had no storage time (*n*=8, 38%). Most participants stored the DNA at room temperature (*n*=12, 57%), while the rest stored the DNA at either −20 °C (*n*=4, 19%) or 4 °C (*n*=4, 19%). For DNA extraction, 13 of the 21 laboratories used a manual extraction procedure while seven used an automatic procedure and one laboratory used both. For both manual and automatic procedures half (7/14 and 4/8) had modifications to the kit/robot protocol.

The DNA concentration (ng µl^–1^) prior to library preparation was measured by most laboratories using a Qubit (*n*=14, 67%). The remaining laboratories used either Nanodrop (*n*=3, 14 % for bacterial cultures and *n*=2, 10 % for DNA) or one of several other methods (*n*=4, 19%).

The DNA quality (e.g. RIN, 260/280 ratio and/or 260/230 ratio) of cultures and DNA prior to library preparation was not measured by almost half (*n*=9/21, 43 %). It is worth noticing that the majority of these (*n*=7/9) used a Qubit for measuring concentration. The most popular choice of those that did measure quality was the Nanodrop (*n*=6, 29 % for bacterial cultures and *n*=5, 24 % for DNA). DNA quality can be further checked by gel electrophoresis, but the majority (*n*=16/21, 76 %) did not apply this approach.

All laboratories used commercial kits for library preparation and most (*n*=19/21, 90 %) used a kit from Illumina. The most popular kits were the Nextera XT (*n*=9/21, 43 %) and the Illumina DNA Prep Kit (previously named Nextera DNA Prep Flex Kit) (*n*=8/20, 38 %).

The genomic DNA was prepared for paired-end sequencing by all except one (*n*=20/21, 95 %) laboratory, which prepared the DNA for single-end sequencing.

All laboratories used an Illumina sequencing platform, ranging from MiSeq (*n*=14/21, 67%), NovaSEQ6000 (*n*=3/21, 14%), NextSeq500 (*n*=2/21, 10%), MiniSeq (*n*=1/21, 5%) and Hiseq 1000 (*n*=1/62, 2%). All details are provided in Table S1.

Preliminary exclusion of data in total, eight laboratories had isolates disqualified due to the resulting genome size that was either more than 105 % or less than 95 % compared to the reference. Six laboratories obtained too large genome sizes for nine isolates (max=135.88 %, min=105.23 %), as opposed to two laboratories with too small genome sizes for four isolates (max=94.01 %, min=85.72 %). Of the 13 failed isolates, five were *S. enterica *, five were *E. coli* and three were *C. coli *. For three of the failed isolates, the multilocus sequencing typing (MLST) could also not be successfully predicted. Two of these three isolates were smaller than expected and their allele profile unable to be identified by MLST analysis. The third, a genome of GPT2020-003 (BACT), was 130 % of expected genome size (Fig. 1d and Table 3). Isolates of GPT2020-005 was completely excluded due to findings in the matrix of SNP distances to reference genome (Table S2).

The preliminary exclusion of data reduced the number of assessed genomes from 223 to 177 encompassing 37 GPT2020-001 genomes (18 BACT, 19 DNA), 38 GPT2020-002 genomes (19 BACT, 19 DNA), 35 GPT2020-003 genomes (16 BACT, 19 DNA), 39 GPT2020-004 genomes (20 BACT, 19 DNA) and 28 GPT2020-006 genomes (11 BACT, 17 DNA).

### Assessment of SNP distance to reference genome

The raw reads of both the cultures (BACT) and corresponding DNA of the six test strains were mapped to the corresponding references to identify SNPs. In most cases, the SNP differences were observed in sequences originating from the cultures (BACT). Differences greater than 10 SNPs were observed in genomes submitted for BACT samples of GPT2020-002 (singleton of 11 SNPs by user 17) and in GPT2020-006 [six of the 28 submissions by User-3 (*n*=1), User-6 (*n*=1), User-10 (*n*=2), User-17 (*n*=1) and User-20 (*n*=1) had more SNPs (min=11, max=16)]. All detected SNP differences are provided in Table S2.

### Assessment of physical coverage to reference chromosome

Genome submissions found to be outliers and below the adjusted quality threshold were found for five laboratories. The percentage of physical coverage was generally high among participants (above 99%), meaning even small deviations could result in the sequence being an outlier. Seven genomes from User-5 (*n*=2), User-08 (*n*=1), User-09 (*n*=2), User-14 (*n*=1), and User-20 (*n*=1) covering all three species [four from culture (BACT), three from purified DNA] were identified as being outliers, making any reliance on sample type or species unlikely.

### Assessment of reads mapping

The performance of the laboratories was also assessed based on the percentage of reads mapping to the closed reference genomes. Two laboratories submitted genomes below the adjusted quality threshold in this metric; User-10 by five out of the six submitted genomes [(BACT, *n*=2), (DNA, *n*=3)] all of which were outliers and User-09 by one out of eight genomes (DNA, *n*=1), which was not an outlier.

### Assessment of the assembly size compared to reference

Assembly size and size compared to reference genome are essentially the same metric and were therefore considered as one. Two laboratories were identified having genomes below the adjusted quality threshold in this metric: User-05 in two of 10 genomes (DNA, *n*=2), one of them being an outlier and User-02 with seven out of eight genomes [(BACT, *n*=4), (DNA, *n*=3)], five of which were also outliers, all exclusively above the adjusted quality threshold and deviating by 1–3 % from the expected size. This corresponds roughly to 50000–150000 bp more than the reference. No *C. coli* samples extended above the threshold, but both purified DNA and culture (BACT) of *S. enterica* and *E. coli* were among the failing genomes. Samples deviating more than 5 % were disregarded, see previous exclusion criteria.

### Assessment of base call accuracy

The probability in base call accuracy, known as *Phred* quality score or Q-score, was assessed and samples were determined as outliers if they were below the adjusted 3SD level. User-10 had five out of six genomes classed as outliers [(BACT, *n*=2), (DNA, *n*=3)]. The quality of these however, were above Q30, which is generally regarded as high confidence in the individual base calls. While this metric is not indicative of the overall quality as found in the linear regression analysis, it is expected to potentially impact the SNP distance matrix. The quality of reverse reads was found to be less than average of the corresponding forward read. However, this is an expected phenomenon for Illumina platforms and does not necessarily indicate a performance issue [17].

### Assessment of insert size

Average insert size is the average distance between paired-end adapters, estimated by BWA-MEM. One participant, User-10, was identified having submitted three of six genomes with an average insert size below the adjusted quality threshold ranging 135.44–147.72 bp for GPT2020-003 (BACT), GPT2020-004 (BACT) and GPT2020-006 (DNA), but without being termed outliers. While this does not necessarily translate to poor sequence quality, insert size was observed to correlate weakly to several other QC metrics, including number of contigs >200 bp and assembly size.

### Assessment of number of contigs

When assessing the number of contigs above 200 bp, two laboratories provided outlying results; User-02 were below the adjusted quality threshold in three of eight genomes [(BACT, *n*=2), (DNA *n*=1)], one of which was an outlier, and User-05 in two of 10 genomes of which both were outliers.

With the same laboratories identified with genomes outlying in the metric ‘Total number of contigs’; GPT2020-004 (DNA) from User-02 and GPT2020-001 (DNA) and GPT2020-002 (DNA) from User-05. The two QC metrics, ‘total number of contigs’ and ‘number of contigs >200 bp’, were found to be strongly correlated across all sample types and species. The same laboratories were identified as having results outlying in the assembly size compared to the reference, which likewise corresponds to their correlation in the linear regression analysis.

### Assessment of N50/NG50

In the assessment of the N50 and NG50, the same approach was used to identify genomes below the adjusted quality threshold. Due to the strong correlation between N50 and NG50, failing either metric was counted as failing the adjusted quality threshold. Just one participant, User-10, had one such genome GPT2020-003 (DNA) in NG50.

### Assessment of sequencing depth

The sequencing depth of total DNA and chromosome was found to correlate with the yields (number of reads, number of reads after trimming, reads mapped to reference DNA and chromosome, unmapped reads). As such, it was not found to impact sequence quality, presumably due to saturation of the physical coverage of the genome. No outliers or values below the adjusted quality threshold were identified for this metric.

### Assessment of the linear regression and quality metrics

From the linear regression, we observed that the number of contigs >200 bp (as well as the total number of contigs) showed a strong positive correlation with the size of the assembled genome and proportion of assembly to reference genome (Fig. 1a). It likewise showed a weak negative correlation to the physical coverage of the reference chromosome and weak positive correlation with the average insert size. N50 showed correlation with physical coverage of the reference chromosome and the proportion of reads mapped to the reference DNA (Fig. 1c). The metrics number of reads, number of trimmed reads, number of unmapped reads, reads mapped to the reference DNA/chromosome and depth of coverage of the reference DNA/chromosome unsurprisingly showed high correlation, but provided little to no information on the overall quality of the sequencing (Fig. 1b). Thus, the general laboratory underperformance was based on producing genomes below the adjusted quality threshold in one of the correlated metrics ‘N50/NG50’, ‘physical coverage of the reference chromosome’ or ‘percentage of reads mapped to the reference genome’ AND one of the correlated metrics ‘number of contigs >200 bp’, ‘average insert size’ or ‘size of the assembled genome’ (Table 3).

### Identification of general laboratory underperformance

The ability to obtain a consistently good quality of sequence data was assessed on the basis of the correlation statistics and linear regression of a number of QC metrics (Table 2). A total of 13 laboratories submitted genomes, which were considered below the adjusted quality threshold in one or more of the assessed QC metrics for at least one of the 10 samples, discounting GPT2020-005 due to SNP-analysis (Table 3). Furthermore, eight laboratories submitted reads, which produced assemblies deviating more than 5 % from the expected genome size. (* S. enterica* (*n*=5), *E. coli* (*n*=5) and *C. coli* (*n*=3)) from User-02, User-06, User-09 User-10, User-12, User-14, User-16 and User-19. In nine out of 13 of these genomes (GPT2020-001 BACT, *n*=1), (GPT2020-002 BACT, *n*=2), (GPT2020-002 DNA, *n*=1), (GPT2020-003 BACT, *n*=4), (GPT2020-006 BACT, *n*=1) the deviation was more than 10%, the specific reasons for which should be clarified.

Only two laboratories, however, were termed as general laboratory underperformers namely User-05 and User-10, who need to assess their WGS workflows and identify the root cause of their insufficient genomes of consistently good quality.

### Contamination estimation with Kraken2

All isolates from User-02 were found to contain a higher proportion of reads called from the genera *Ralstonia* (0.08–0.23 %)*, Variovorax* (0.03–0.10 %) and *Mycobacterium* (0.0–0.26 %) than other participants. User-05 isolates contained comparably high levels of *Listeria* (0.14–0.33 %) in samples excluding GPT2020-006-DNA and *E. coli* and *S. enterica* samples were found to contain comparably high proportions of *Campylobacter* reads (0.08–0.36 %). User-09 and User-10 had a higher number of unclassified reads at 0.06–2.66 % and 0.78–2.42 %, respectively. User-14 showed slight contamination by *Listeria* in all samples (0.03–0.15 %). Multiple users showed some degree of contamination by *Homo sapiens*. Kraken2 results are summarized in the supplementary material.

### Quality-control thresholds

Setting overall QC thresholds indicating good sequencing quality for *S. enterica, E. coli* and *C. coli *, was possible for the QC metric ‘number of contigs >200 bp’ and ‘N50’. Thus, suggesting QC thresholds for N50 being greater than approximately 20 000 and 25 000 for *C. coli* and *E. coli *, respectively, and a threshold for number of contigs >200 bp lower than 390, 495 and 205 contigs for *S. enterica *, *E. coli* and *C. coli,* respectively.

The QC thresholds indicating good sequencing quality of ‘N50’ for *S. enterica* was not possible due to a high SD level resulting in negative values when calculating the lower 3SD value. QC thresholds for ‘N50’ were, however, possible to set using the lower 3SD based on the average of the remaining C. coli isolates (GPT2020-006 BACT and DNA) and *E. coli* (GPT2020-003 and GPT2020-004, both BACT and DNA). Looking at the distribution of the ‘number of contigs’ for each sample, it is clear they do not follow a standard normal distribution. As values cannot be negative, the distribution is better described as a truncated or half-normal distribution. Correcting SD and mean for a half-normal distribution gives an average upper threshold for 3SD above the mean of 225, 265 and 100 ‘contigs’ for *S. enterica *, *E. coli* and *C. coli *.

Statistical tests did not show significant difference between the DNA and BACT of the same isolate in neither N50 nor number of contigs >200 bp.

## Discussion

The GPT2020 was successfully organized based on the concept of the past GMI PT. It was offered to the EURL AR network with the objectives to assess the participating laboratories ability to obtain consistently good quality WGS data based on several QC WGS metrics, SNP analysis and MLST prediction as well as to identify underperforming laboratories in need of assistance.

Most laboratories performed well and only two laboratories were identified as overall underperforming (User-10 and User-05), having serious sequencing problems with one or more of the up to 12 submitted genomes. The GPT2020 highlights the importance and impact of comparing laboratories’ results to access each laboratory individually and confirm the quality of their performance, which is essential for QC. Specifically, laboratories for which sequence data generation is an accredited method under ISO/IEC 17025 : 2017 are required to perform regular checks on the quality of their results and performance, this includes participation in proficiency tests or interlaboratory comparisons [18].

To identify underperformance, the QC metrics in combination allowed for identification of laboratories determined as overall underperforming. The ‘percentage of physical coverage’, ‘percentage of reads mapped to the reference genome’ and the ‘genome size compared to the reference genomes’, were not suitable for routine QC assessment which would require a genome of the exact same strain being assessed. For a proficiency test however, they are valuable metrics. The correlated statistics, support the choice of N50 and number of contigs >200 bp being suitable QC metrics for routine QC assessment of sequencing quality as indicated above.

In brief, User-10 submitted a genome (GPT2020-003 DNA), which was below the adjusted quality threshold for NG50. This is perhaps due to filtering out submissions, which did not satisfy less than 5 % deviation from the expected size, which eliminated four of the participants’ 10 submitted genomes. This removed not only suspected contaminations, but poor sequencing runs as well. User-10 submitted *C. coli* genomes, which showed comparably more SNPs in the SNP distance matrix, which could be expected to improve with high enough sequencing depth. User-10, however, achieved considerable depth (GPT2020-006 DNA 223 x and BACT 136 x). More likely the issue is caused by the same mechanism causing their consistent poor mapping performance. While User-10 had acceptable *Q*-scores, they were low comparatively to the other participants, perhaps indicating over-clustering in their runs. Likewise, it is possible that the relatively short insert size impacted the subsequent assembly and mapping performance. They were the sole user reporting the use of Kingfisher extraction system and Magmax core purification kit. Considering their number of contaminated genomes, residual contamination of isolates or reagents could be a factor. We recommend eliminating risks of residual contamination of reagents and isolates and revisiting library preparation workflow to identify potential risks to the integrity of their isolate and reagents purity.

User-05, had GPT2020-001 DNA and GPT2020-002 DNA as outliers in the metrics ‘size of genome size compared to the reference’ and ‘number of contigs >200 bp’, as well as GPT2020-001 BACT and GPT2020-004 BACT as outliers in ‘physical coverage of the reference chromosome’. This flags the participant in the two uncorrelated statistic groups. As issues in size and contigs from *S. enterica* genomes stem from purified DNA, storage or handling of the sample could be speculated to cause DNA degradation, which would impact the assembly. It was observed that User-05 generally had a high number of contigs among participants, as apparent from the boxplots (Fig. S1). The lack in coverage of the reference chromosome was minute, ranging from 0.02–0.12 % attesting to comprehensive coverage among participants. Kraken2 estimates a point towards possible contamination by *Campylobacter* and *Listeria *. Our recommendation is to reconsider current storage and in-lab handling practices and if possible, shipping and storage conditions to avoid excessive DNA breakage or contamination.

Although harmonization is desirable to facilitate easy comparison, it poses some risk. It has been observed that the Nextera XT kit performs poorer in species and sequences with low GC content [19, 20]. Meaning wide usage could introduce systematic issues for surveillance of priority pathogens such as *Campylobacter *. Furthermore, it has disadvantages such as creating a monopoly for a limited number of sequencing platforms and consumable producers. This could be a huge barrier for further developments of the technology, but more importantly hinder a competitive market in reducing the WGS costs to further allow the technology to reach LMICs, which have the potential to leapfrog the technology. The recent advent of long-read technologies such as Oxford Nanopore (ONT) have improved these prospects considerably, due to the limited costs of equipment compared to the massive investment of benchtop platforms. Despite this, reagents may still pose a possible challenge in terms of both acquisition and cost.

In the assessment of QC metrics for evaluating performance, several metrics were found to be correlated with widely used metrics ‘N50’ and ‘number of contigs >200 bp’. The ‘N50’ metric correlated well with ‘percentage of physical coverage’ and ‘percentage of reads mapped to the reference genome’. The latter two, however, are ill suited for routine sequencing, as they rely on a reference genome. In terms of the physical coverage, near 100 % coverage was observed across all genomes that passed initial exclusion based on size compared to the reference. This is likely, due to the exclusion of genomes less than 95 % of the reference genome size. As such, no QC-metric other than N50 was found to estimate completeness of the sequencing in strains without a reference genome, though it was observed that for half (*n*=2) of the genomes below the 95 % size threshold, the MLST was not determined. Including a routine check of select housekeeping genes after species verification could be a useful secondary verification to ensure the full capture of the genome. The proportion of reads mapped to the reference genome could perhaps be used in an alternative form, by measuring the percentage of reads mapping to the longest N contigs or similar. The metric is important as a high proportion of unmapped reads means more wasted resources.

The ‘number of contigs >200 bp’ was found to correlate with the ‘average insert size’ and ‘percentage in size compared to the reference genome’. Laboratories submitting data below the adjusted quality threshold in ‘average insert size’, were not observed to have data below the adjusted quality threshold of the genome size and the number of contigs. Perhaps again due to initial removal of submission, which deviated more than 5 % on genome size. Average insert size can be used despite lacking a reference genome, though its effect has been reported to be negligible on reads longer than 250 bp [21]. In this assessment, we looked at a range from 114 to 522 bp, which may explain why it was observed to have any impact at all. As longer insert size is expected to contribute to more complete assembly, it is less obvious if increased insert size results in overestimation of the genome size, as we here found a positive correlation between insert size and genome size. In genomes like *E. coli *, where there can be large differences between lineages, even 10 % above the actual genome size might not be detectable. The reason for this increase in size should be further investigated to determine whether it is a result of contamination, error in the workflow or artefacts from the bioinformatics methodology.

In relation to the SNP analysis, it is difficult to determine whether the imposed threshold of 10 SNPs was adequate. In the *spp*. of *E. coli* and *S. enterica *, each had one genome with relatively few SNPs called from submitted genomes (less than three) and one genome with comparably more, ranging from one to 11 SNPs. An explanation could be due to error in the reference genome, or SNPs being called from a hypervariable position [22]. While steps are taken to avoid the latter by excluding regions with SNPs in close proximity, perhaps the near clonality of the genomes results in such regions being included, leading to more SNPs being called. Perhaps a reference genome can likewise increase the number of SNPs as reads may be erroneously mapped to replicate regions. Taking into consideration the number of pairwise SNPs between submitted strains rather than to the reference may be a solution, as both *E. coli* and *S. enterica* had less than five SNPs between submitted isolates. This approach is also closer to what is possible when surveilling for new outbreaks in an industrial setting, where a reference is unlikely to be available. The *C. coli* GPT2020-006, however, generally showed larger number of SNPs, despite its smaller size. Using a different threshold for this species may be prudent but may also be influenced by the bias introduced by library preparation kits in *spp*. with low GC content discussed previously [20]. As GPT2020-005 was excluded from the dataset and less isolates for GPT2020-006 were submitted than for *S. enterica* (GPT2020-001- GPT2020-002) and *E. coli* (GPT2020-003 – GPT2020-004), the results for *C. coli* are not as well supported.

A general tendency was observed for the reverse reads to have a lower quality score than the forward reads, in accordance with previous findings, [17, 21], and is likely explained by the initial additional rounds of synthesis. Additionally, multiple participants were found to have sequences contaminated with human DNA, but it appears to have little effect on QC even when constituting more than 1 %.

Several metrics on reads and sequencing depth was likewise evaluated, and all found to strongly correlate: number of reads, number of trimmed reads, number of reads mapped to reference DNA/chromosome and depth of reference DNA/chromosome, however they were found to have little effect on overall quality. Perhaps due to a saturation of coverage of the genome, which of course is the goal of the WGS analysis. It was observed that some participants had a very high sequencing depth (above ×500), which is unnecessary and indeed impedes analysis, as this redundant information demands more computing power. While depth should be substantial enough for accurate SNP-calling, this level of redundancy for single isolates is wasteful and costly.

Another goal of the GPT2020 was to set QC thresholds for several parameters, which will allow for future self-evaluation of WGS data by laboratories daily. Currently, no overall international standardization exists as to which WGS QC metrics should be assess prior to completing a sequencing run as well as the level of thresholds for such metrics. Despite the lack of the international standardization, several organisations and supranational entities have issued or are in the preparation of drafting guidelines describing QC metrics and QC thresholds levels. In the current versions of the ISO 23418 : 2022. Whole-genome sequencing for typing and genomic characterization [23], the EFSA statement on the requirements for WGS analysis of micro-organisms intentionally used in the food chain [24], the report from the EUCAST subcommittee about the role of WGS in antimicrobial susceptibility testing of bacteria by Ellington *et al*. [25], and the EURL AR WGS protocol (https://www.eurl-ar.eu/wgs.aspx), the same QC metrics as evaluated in this GPT2020 has been suggested. Currently, the thresholds levels of the QC metrics are not precise. The version of the ISO 23418 : 2022 standard does not contain thresholds QC levels, whereas the EFSA statement only indicates that the number of contigs for bacteria should be below 500 and that the total length of an assembly should not exceed +/-20 % of the expected genome size [25] suggests that a minimum depth of 30X is preferred, the total number of contigs below 100 is realistic for organisms with genomes size of 5 to 6 Mb but otherwise below 1000 indicates good quality, and a N50 larger than 15 000 normally indicates good quality [25]. These QC thresholds for N50 and number of contigs, correspond well with what was observed in this GPT. Using 3SD from the mean corrected for half-normal distribution, we set 265 contigs for *E. coli *, 100 contigs for *C. coli* and 225 for *S. enterica* as QC threshold levels. Here we have used contigs >200 bp, which will inflate to number of contigs compared to the suggested >500 bp. This threshold assumes that the distribution approximates a half-normal distribution, though a truncated normal distribution is perhaps more likely. The theoretical lower limit for number of contigs is the chromosome plus number of plasmids, but for short reads the practical limit is most likely below 100 for high-quality data. A normal distribution is assumed, but this might not be descriptive of the true distribution, and it will in any case be limited by the number of samples. The application of 3SD means the data removed was likely from the lower 11 % of the distribution according to Chebyshev’s inequality [26]. While this does possibly remove outstanding performance as well, most of the QC metrics subjected to outlier removal have limits which high-quality data will trend towards. This means high variance is more likely due to low quality. The proposed ‘N50s’ are 5000–1000 bp larger than those previously indicated as acceptable, possibly due to limiting the recommendations to a specific genus. The values found here however, are closer compared to those that have been previously described as obtainable, with some reservation as to *C. coli* as thresholds were set using only GPT2020-006 genomes [21].

We identified a few limitations in the conduct of the GPT2020. The *C. coli* isolates proved particularly difficult largely due to their anaerobic requirements. Several submitted reads not corresponding to the annotation with the reference genome were excluded from further analysis and were considered as sample mix-up or laboratory contamination. Surprisingly, some laboratories were unable to keep track of the GPT2020 samples and keep cultures and DNA pure while performing laboratory work [27]. The 5 % deviation exclusion criteria may have been overly conservative, though most of the excluded samples were found to deviate by more than 10 %. Most of the excluded genomes originated from culture samples (BACT) supporting the hypothesis of poor laboratory procedures were due to contaminations.

## Conclusions

The GPT2020 was in large a great success with most participants able to deliver high-quality sequence data for all species. Only two laboratories out of the 21 participating were identified as overall underperformers, though this does not account for samples excluded due to possible contamination.

Using the high-quality data from participants, we have suggested minimal quality thresholds for ‘N50’ and ‘number of contigs’ in *E. coli *, *C. coli* and *S. enterica *. Reliable WGS data is fundamental for proper surveillance of AMR and identifying emerging outbreaks. The overall improvement observed during the GPTs facilitate a wide European network of pathogenic surveillance across sectors as we move toward wider implementation of the One Health approach. Reliable methodology and data is naturally paramount for effective policy making and intervention. In the future, we expect to improve our evaluation of participant data considering the presented findings and expand upon metrics. One is the inclusion of mobile genetic elements such as plasmids, which play key roles in AMR transmission and are an immediate threat to public health. The WGS approach likewise allows for possible expansion into other areas important in the clinical setting, such as virulence factors or survival genes. Looking into the concordance between genotype and phenotype is likewise a considerable concern, which we expect to investigate further.

## Supplementary Data

Supplementary material 1

Supplementary material 2
