# Supplementary material for: Results of the 2020 Genomic Proficiency Test for the network of European Union Reference Laboratory for Antimicrobial Resistance assessing whole-genome-sequencing capacities
Source: Microb Genom. 2023 Aug 1;9(8):mgen001076. doi: 10.1099/mgen.0.001076 (PMC10483428; doi:10.1099/mgen.0.001076)
Supplement: Supplementary material 1 [file mgen-9-1076-s001.pdf]

Supplementary figure 1: Boxplots of QC parameters

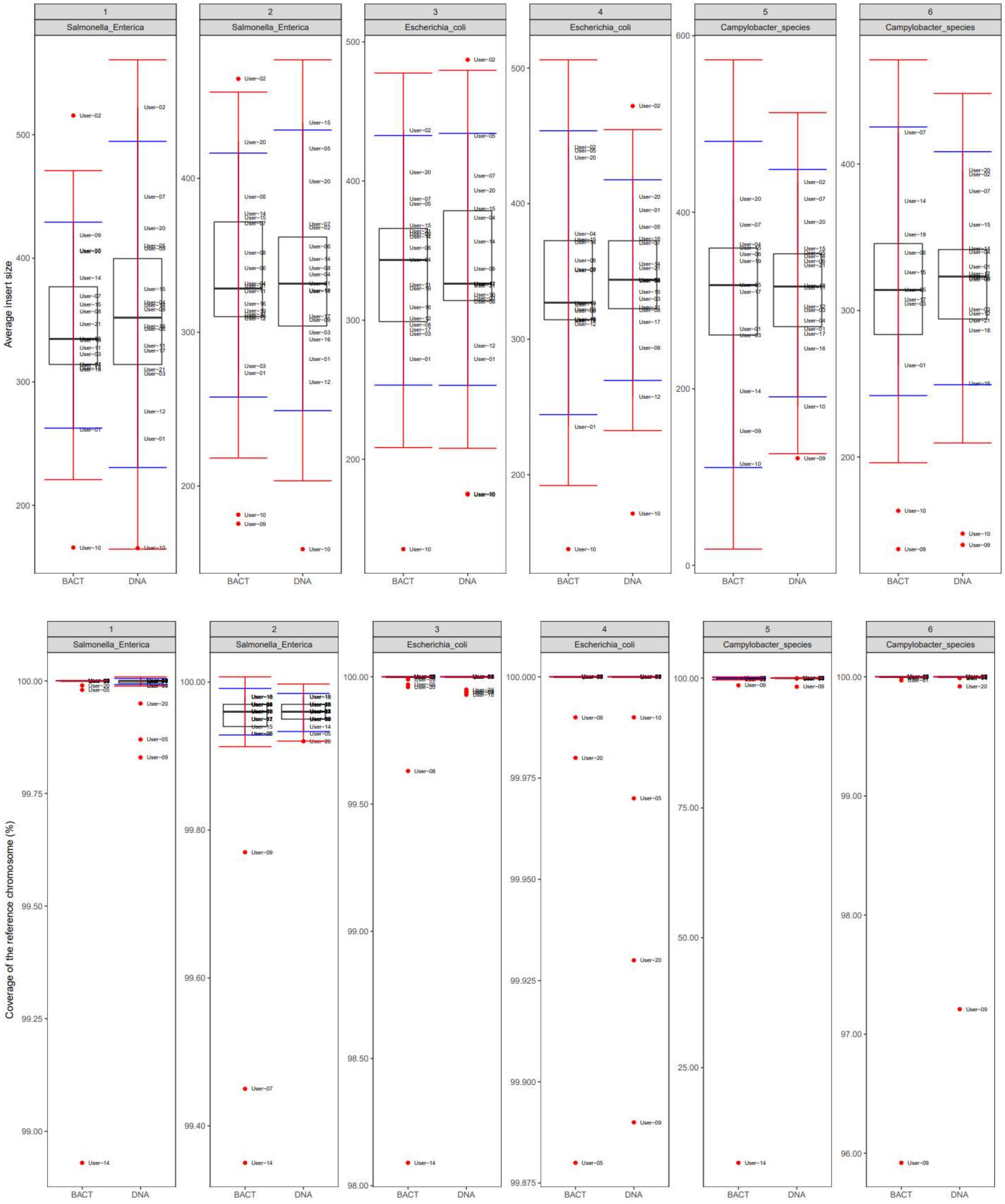

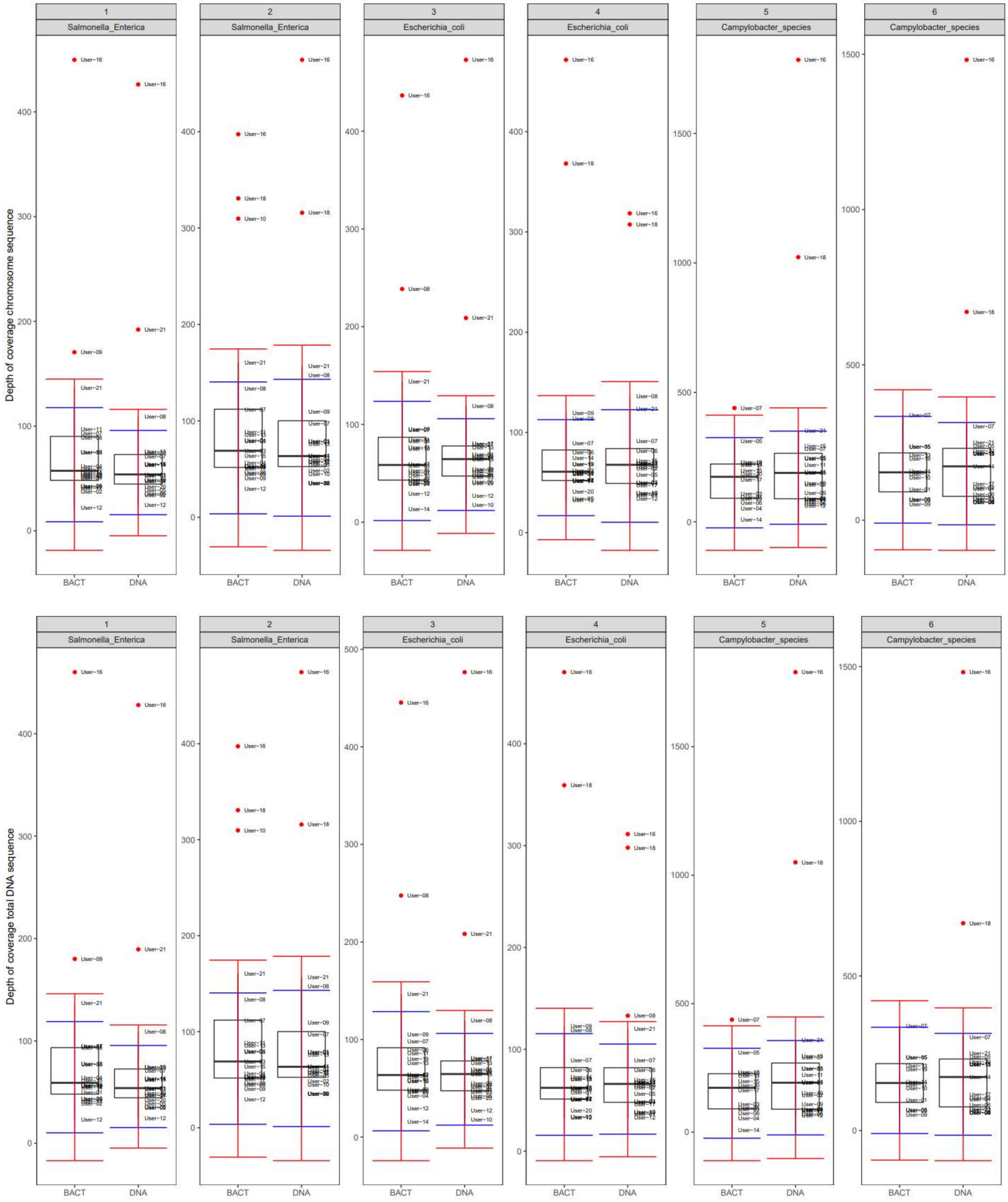
Depth of coverage chromosome sequence
Depth of coverage total DNA sequence
1
Salmonella_Enterica
2
Salmonella_Enterica
3
Escherichia_coli
4
Escherichia_coli
5
Campylobacter_species
6
Campylobacter_species
BACT
DNA
User−16
User−18
User−10
User−09
User−21
User−08
User−07
User−12
User−14

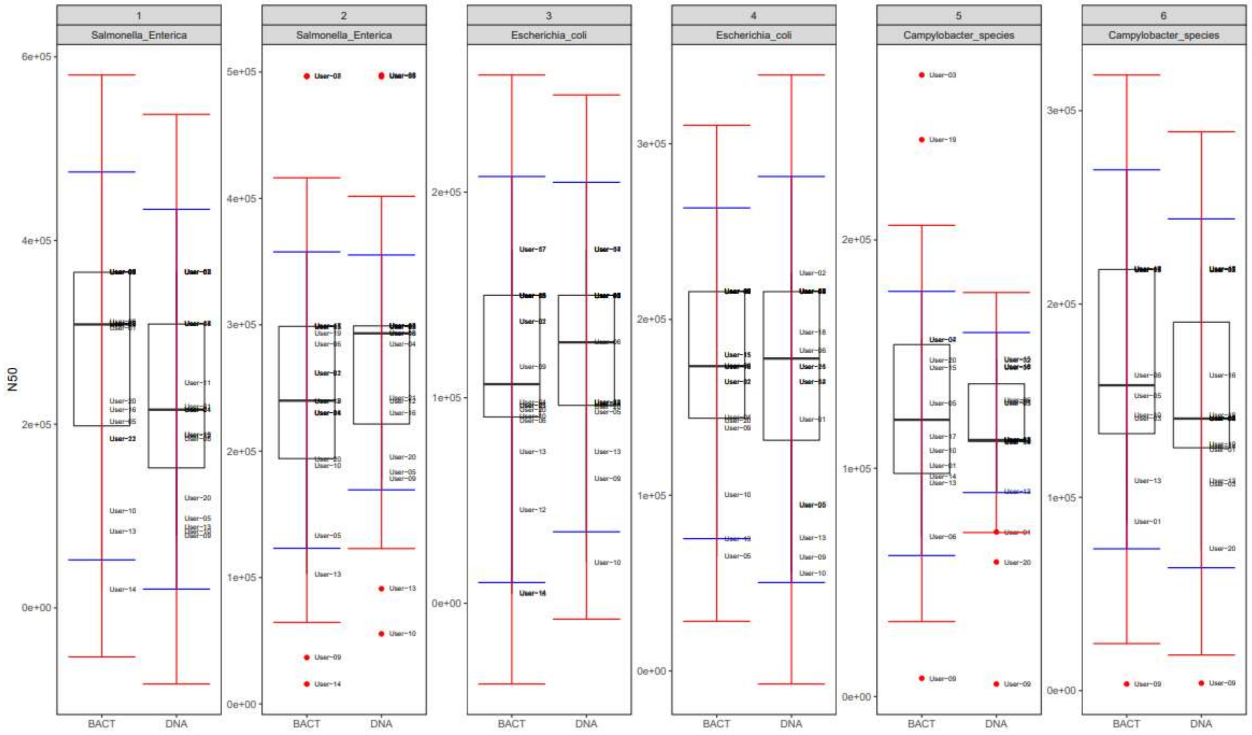
1
Salmonella_Enterica
2
Salmonella_Enterica
3
Escherichia_coli
4
Escherichia_coli
5
Campylobacter_species
6
Campylobacter_species
N50
6e+05
4e+05
2e+05
0e+00
5e+05
3e+05
1e+05
BACT
DNA

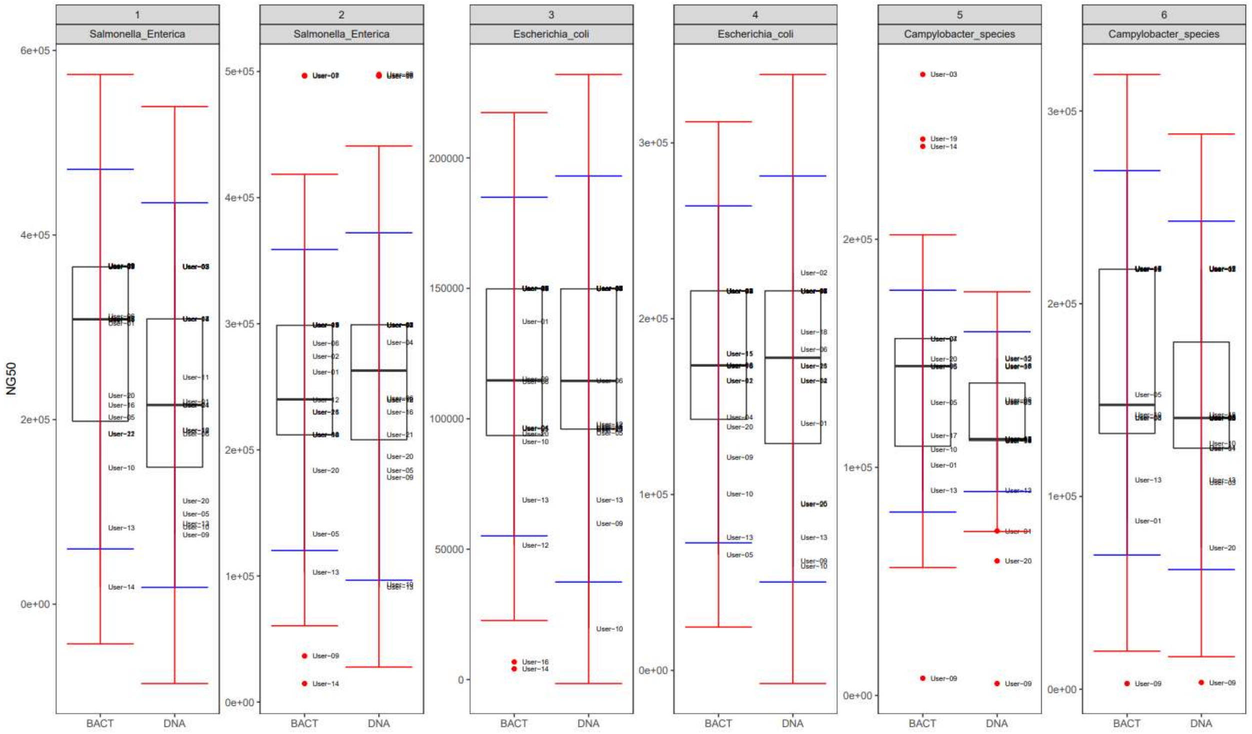
1
Salmonella_Enterica
2
Salmonella_Enterica
3
Escherichia_coli
4
Escherichia_coli
5
Campylobacter_species
6
Campylobacter_species
NG50
6e+05
4e+05
2e+05
0e+00
200000
150000
100000
50000
0
BACT
DNA

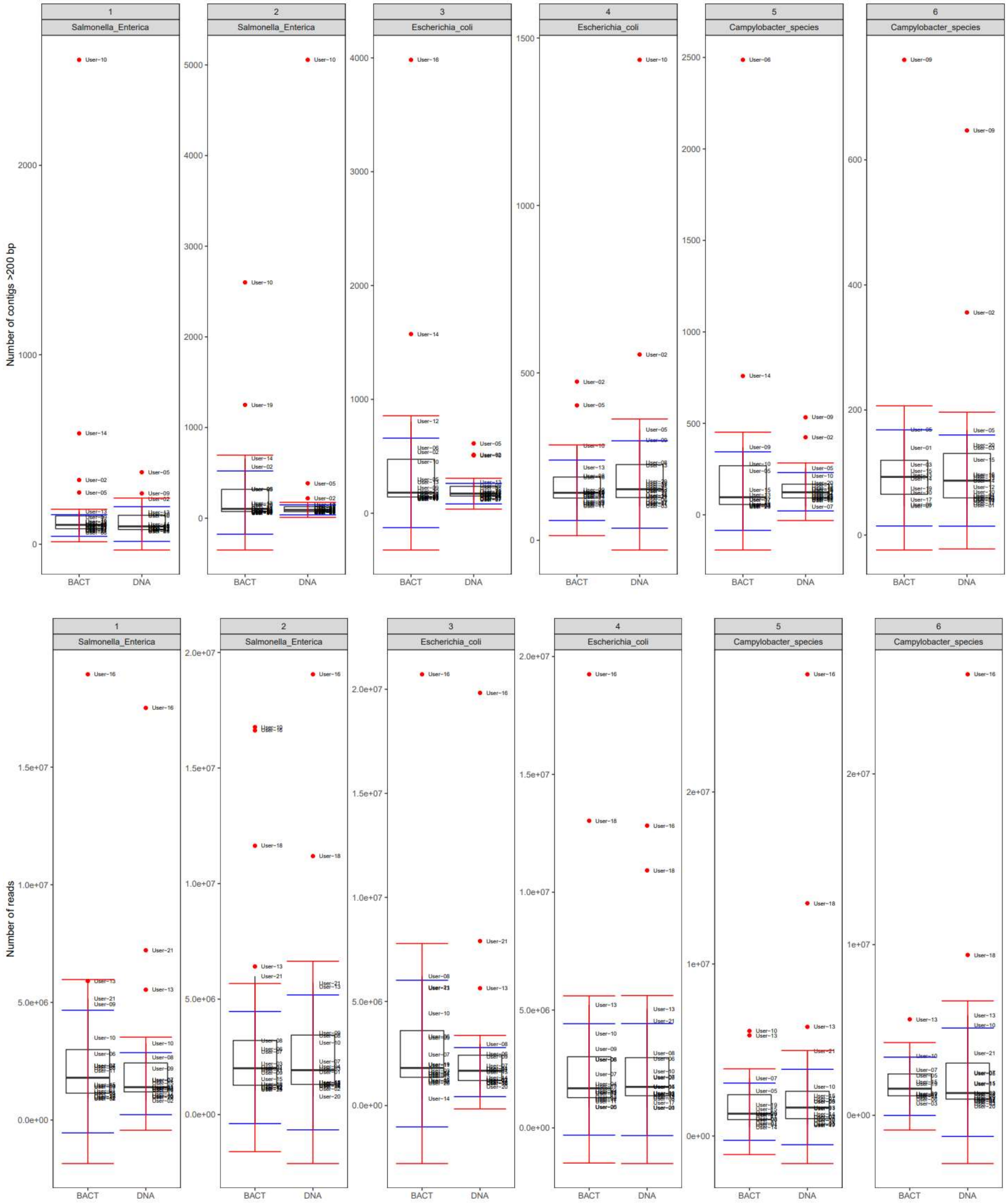

Number of contigs >200 bp
Number of reads
1
2
3
4
5
6
Salmonella_Enterica
Escherichia_coli
Campylobacter_species
BACT
DNA

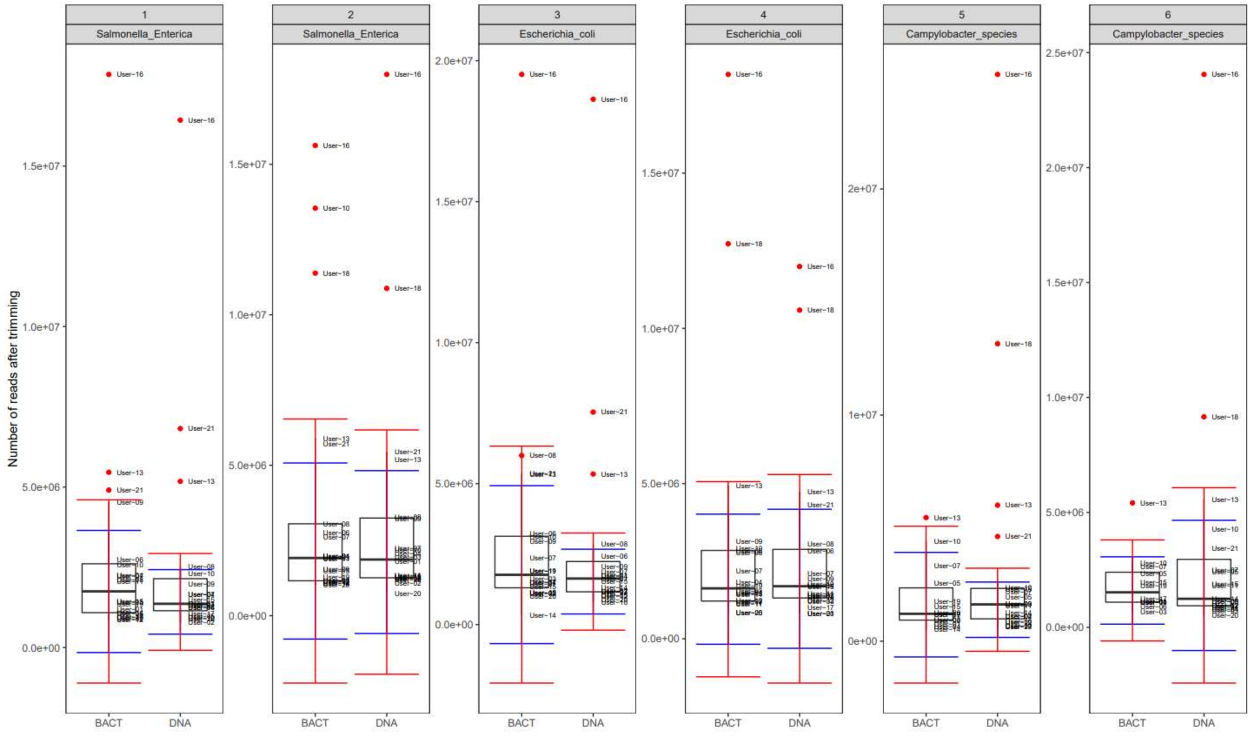
1
Salmonella_Enterica
2
Salmonella_Enterica
3
Escherichia_coli
4
Escherichia_coli
5
Campylobacter_species
6
Campylobacter_species
Number of reads after trimming
BACT
DNA

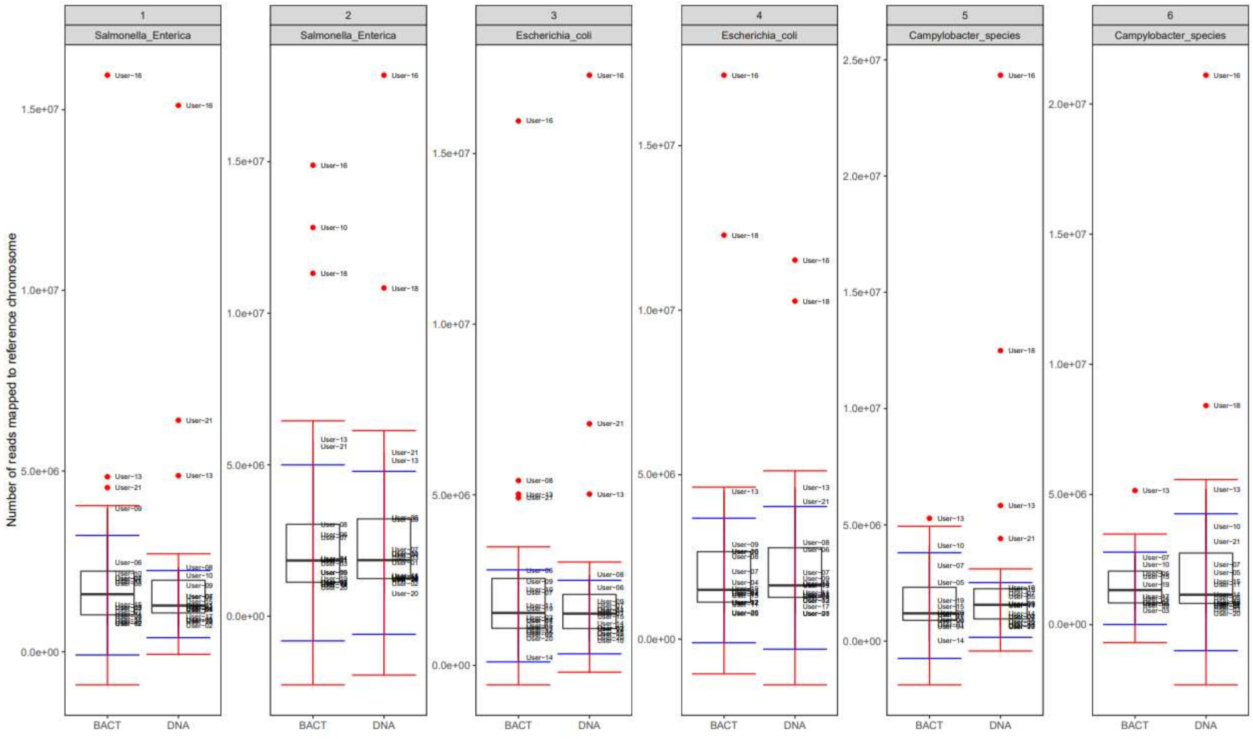
1
Salmonella_Enterica
2
Salmonella_Enterica
3
Escherichia_coli
4
Escherichia_coli
5
Campylobacter_species
6
Campylobacter_species
Number of reads mapped to reference chromosome
BACT
DNA

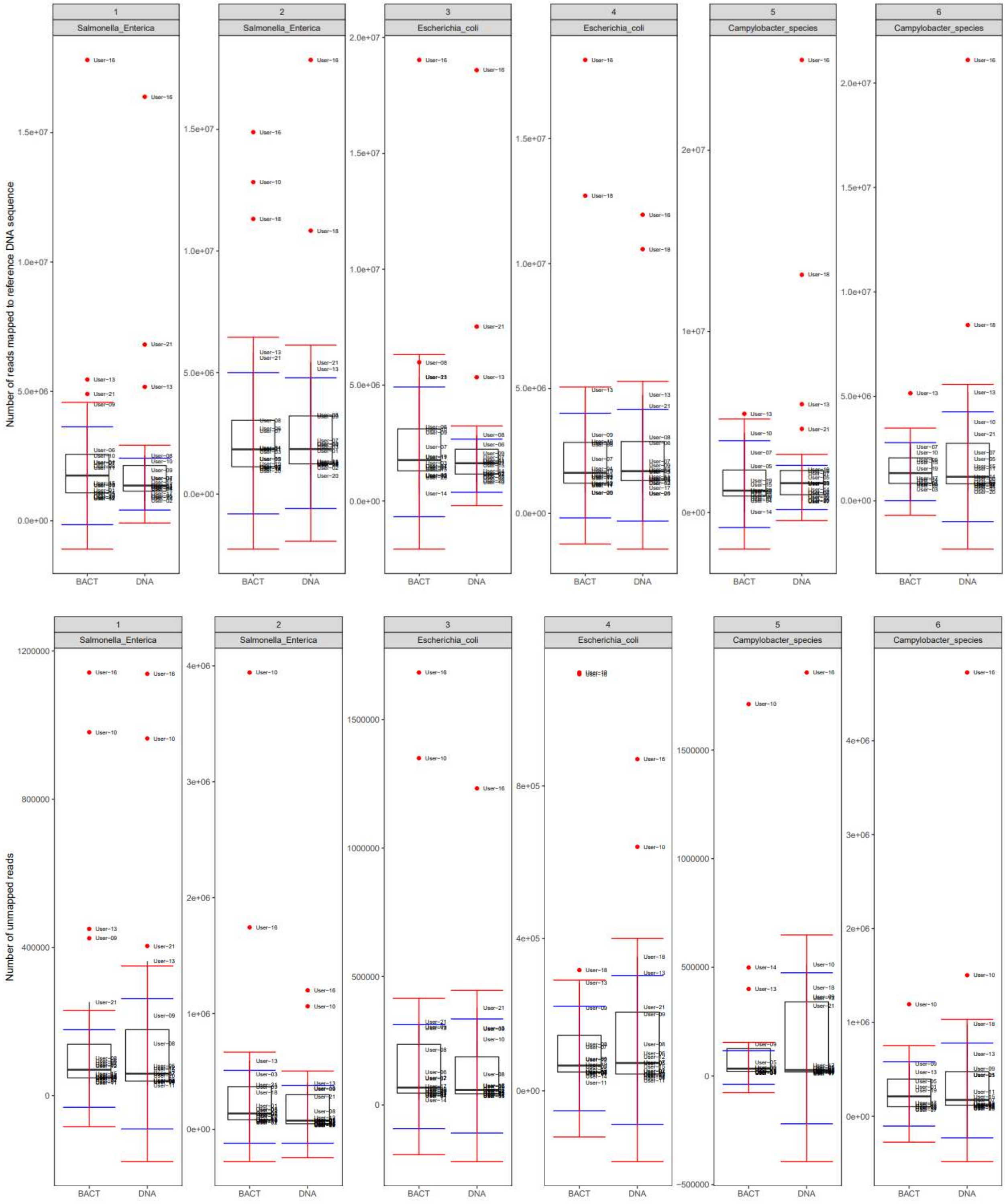
Number of reads mapped to reference DNA sequence
Number of unmapped reads
1
2
3
4
5
6
Salmonella_Enterica
Escherichia_coli
Campylobacter_species
BACT
DNA

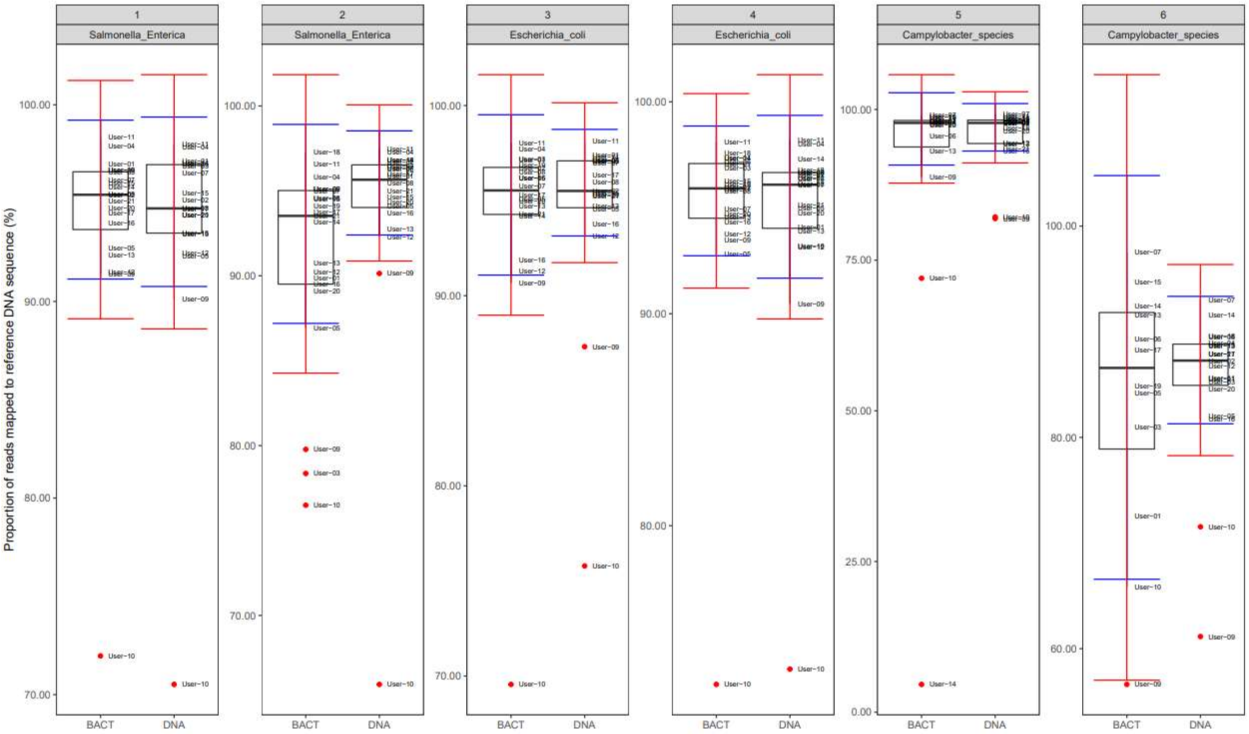
1
Salmonella_Enterica
2
Salmonella_Enterica
3
Escherichia_coli
4
Escherichia_coli
5
Campylobacter_species
6
Campylobacter_species
Proportion of reads mapped to reference DNA sequence (%)
BACT
DNA

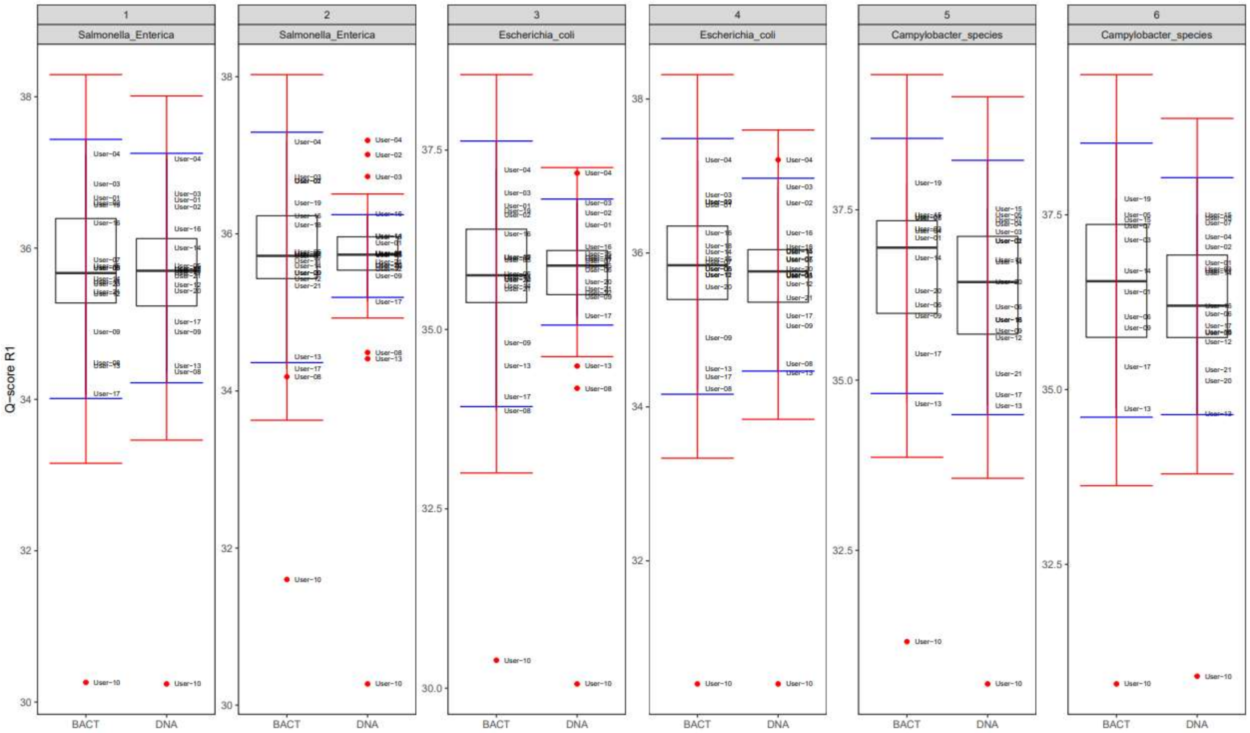
1
Salmonella_Enterica
2
Salmonella_Enterica
3
Escherichia_coli
4
Escherichia_coli
5
Campylobacter_species
6
Campylobacter_species
Q-score R1
BACT
DNA

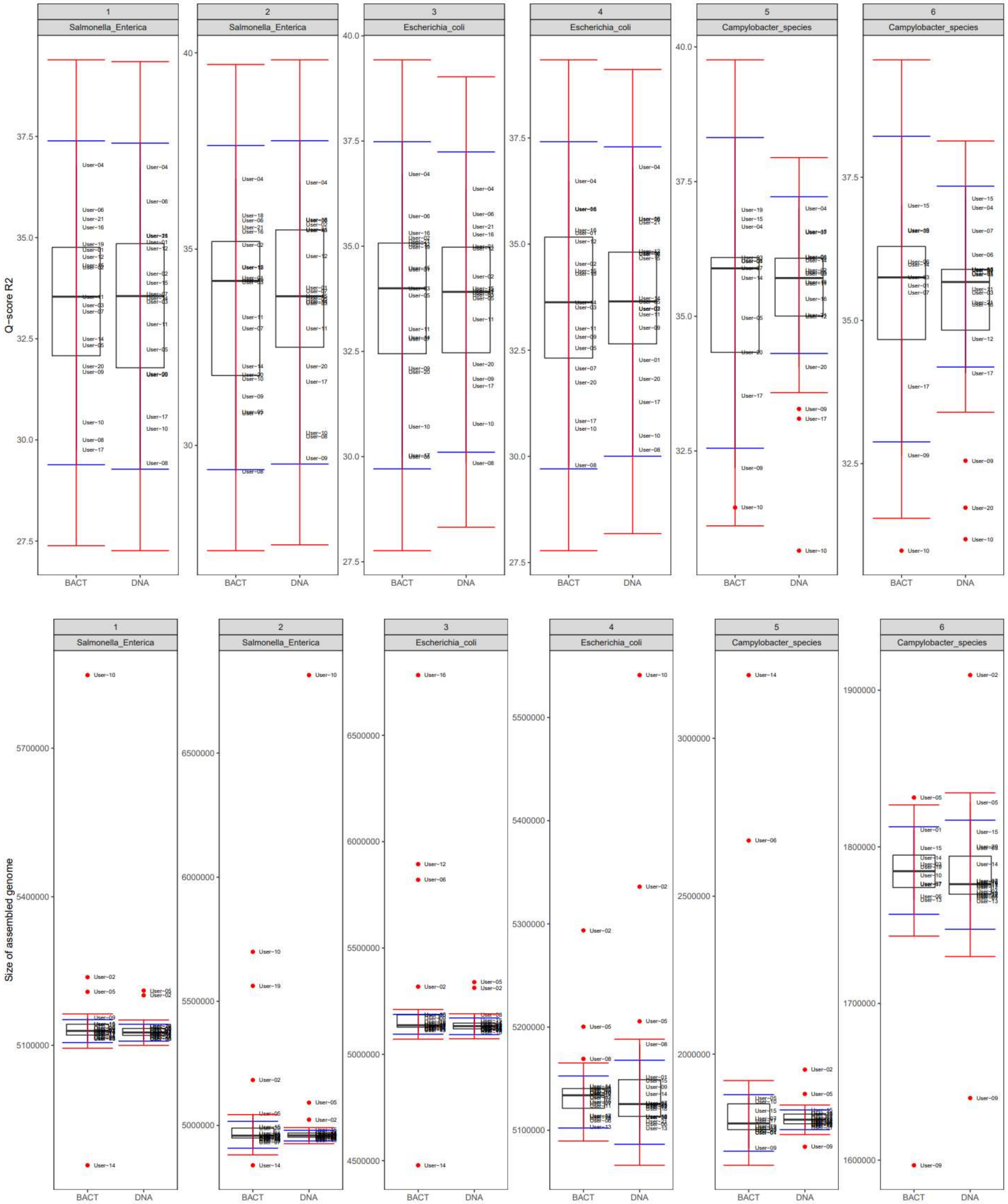

1
Salmonella_Enterica
2
Salmonella_Enterica
3
Escherichia_coli
4
Escherichia_coli
5
Campylobacter_species
6
Campylobacter_species
Q-score R2
BACT
DNA
Size of assembled genome

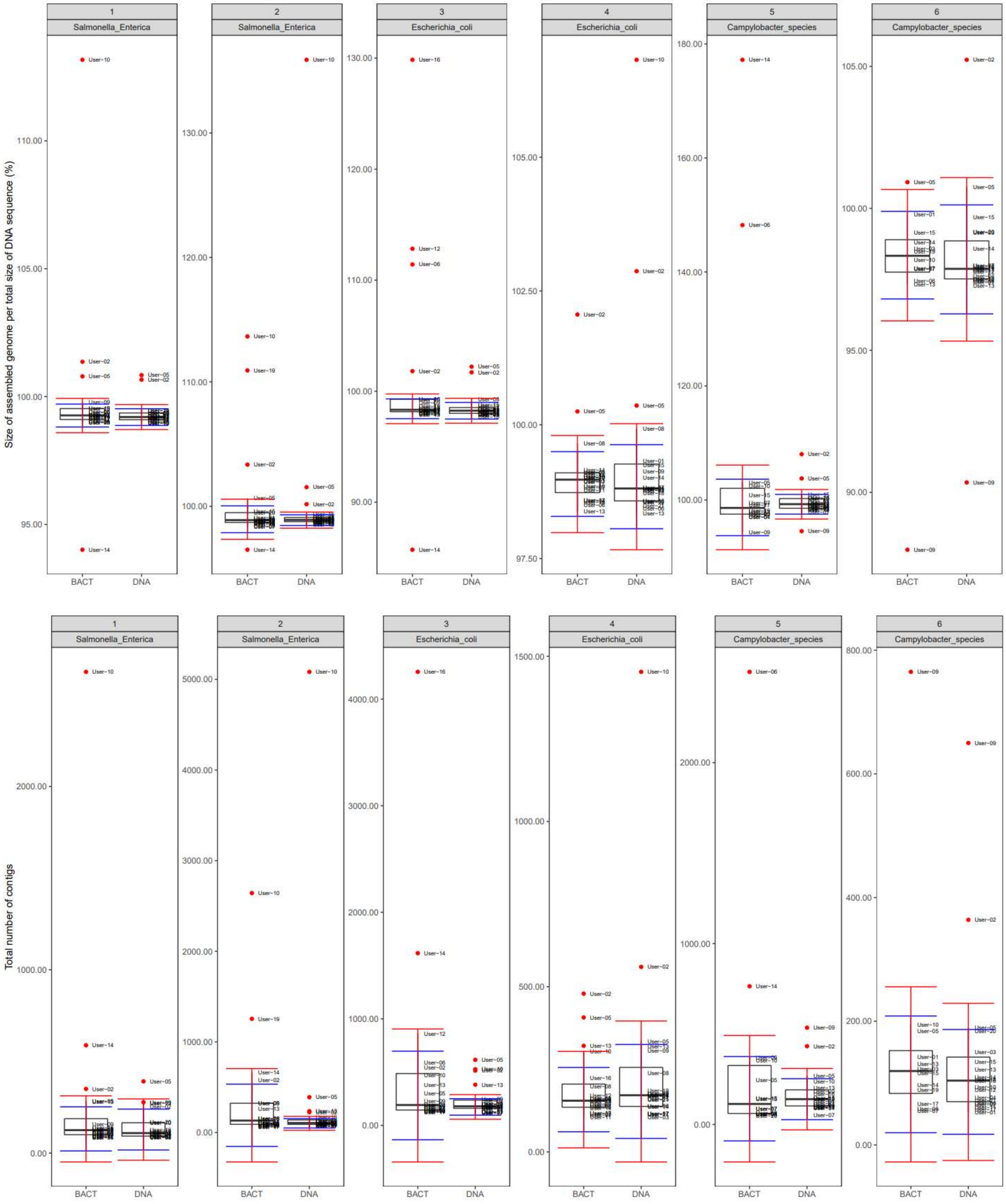

Supplementary figure 1: Boxplots for each of the 12 QC metrics, each isolate segregated into DNA/BACT.

Data points below or above the first and third quartiles, respectively, by more than 1.5 times the

difference between the first and third quartiles, are presented as dots. 2SD and 3SD visualized as blue and red whiskers, respectively.

Supplementary figure 2: Overview of data workflow

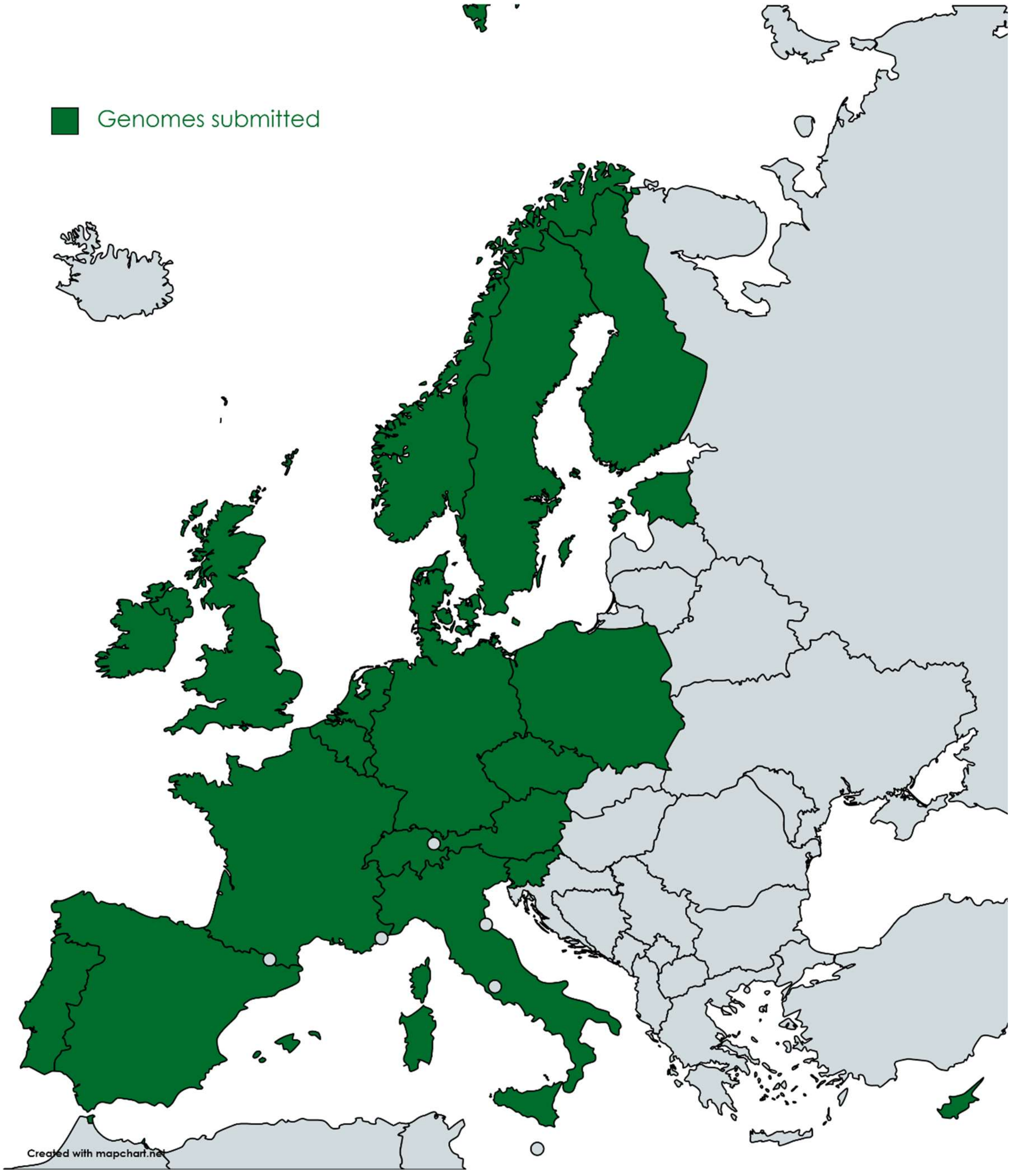

Supplementary figure 2: Map of Europe highlighting participating countries. Made with mapchart.net (https://www.mapchart.net/index.html)

Full Materials and Methods

Organization

The GPT20 was coordinated by the Technical University of Denmark, National Food Institute, Denmark in its capacity as the European Union Reference Laboratory for Antimicrobial Resistance (https://www.eurl-ar.eu/).

Participating laboratories

Participation was offered to the European network of National Reference Laboratories (NRL) for antimicrobial resistance within the food and veterinary sectors. Invitation letters were distributed on the 20th of December 2019 by e-mail to the EURL-AR network to announce participation in the GPT20. Participating laboratories are identified by user codes and are confidential. Participant identity is only known by the corresponding laboratory and the GPT organizers, including the European Commission (EC) and the European Food Safety Authority (EFSA).

Reference strains

Two isolates of three bacterial species were selected for the GPT20, among strains with resistance mechanisms or profiles of particular interest to the EURL-AR network. The selected species for the GPT20 were the following priority pathogens: *Salmonella enterica* (*S. enterica*), *Escherichia coli* (*E. coli*), and *Campylobacter* spp..

Genomic DNA was extracted from the six GPT isolates using an Invitrogen Easy-DNA KitTM (Invitrogen, Carlsbad, CA, United States) and DNA concentrations were determined using the Qubit dsDNA BR assay kit (Invitrogen). The genomic DNA of each strain was divided into two fractions of which one was dried and supplemented with DNA stabilizing agent (DNAstable® Plus, Biomatrica, https://www.biomatrica.com/download/hp-dnastable-plus-handbook/) and sent to the participating laboratories as GPT material as well as being used to provide a closed reference genome. The remaining fraction was prepared for Illumina pair-end sequencing using the Illumina (Illumina, Inc., San Diego, CA, United States) Nextera XT® Guide 150319425031942 following the protocol revision C1. A sample of the pooled Nextera XT Libraries was loaded onto an Illumina NextSeq reagent cartridge using NextSeq Reagent Kit v2. The libraries were sequenced using an Illumina NextSeq platform.

To enable the assessment of the differences in the sequences generated by the laboratories, for each of the GPT strains closed genomes were produced. Multiplexed microbial SMRTbell libraries were prepared using the SMRTbell prep kit 3.0 according to PacBio protocol (PacBio, Menlo Park, CA, November 2021). The multiplexed SMRTbell library was then sequenced on

the Sequel IIe system (PacBio, Menlo Park, CA) using Binding Kit 3.2 and Sequel sequencing kit 2.0 on one SMRT cell 8M (PacBio, Menlo Park, CA), with 30 hours collection time. Raw HiFi reads were demultiplexed by running the Demultiplex Barcodes application and de novo assembled using the Microbial Assembly in SMRTLink (PacBio, Menlo Park, CA). The resulting assemblies were circularized and oriented to start at oriC.

.

The closed reference genomes were analyzed using *in silico* bioinformatics tools to confirm the identify the MLST (Multilocus sequence typing) sequence type (ST) genes using the pipeline; MLST v 2.0.4 available from Center for Genomic Epidemiology http://www.genomicepidemiology.org/

Distribution of reference material

Individual sets of the reference material were distributed by the EURL AR to each participating laboratory on the 22nd of September 2020, along with a cover letter. Each participant received two isolates of each species named (GPT20-001-6 (BACT)), as well as corresponding genomic DNA named (GPT20-001-6 (DNA)). The reference material was distributed in double pack containers (class UN 6.2) according to the International Air Transport Association (IATA) regulations as UN3373, biological substances Category B.

Procedure

Protocols on how to handle the received bacterial cultures and DNA were made available on the Global Surveillance website (https://www.globalsurveillance.eu/projects/genomic-proficiency-test-2020). Additional relevant information was distributed by email directly to the laboratories. Participating laboratories were asked to perform the GPT2020 using WGS methodologies routinely employed at their laboratories.

Furthermore, the participants were requested to capture relevant information via an online survey, which contained questions about the methodology applied. This included information such as the cultivation and DNA extraction procedure, the quality assurance metrics applied, details related to the sequencing and analysis of the obtained sequencing data (Supplementary Table 1)

Sequencing quality analysis

The laboratories were requested to submit their generated WGS raw sequence files in fastq format to a DTU hosted ftp-site. Subsequently, the raw reads were also submitted by the GPT provider to the European Nucleotide Archive (ENA) under project accession number PRJEB58706.

As part of the GPT, these reads were run through an in-house pipeline, where the reads were *de novo* assembled using the SPAdes v 3.9.0 software (Prjibelski et al., 2020). Subsequently, reads were aligned to closed reference genome using Burrows-Wheeler Aligner (BWA)-MEM algorithm v 0.7.12 (Li, 2013) with default settings and Samtools v 1.2 (Li et al., 2009) was used to filter the reads that did not map. Finally, each of the QC metrics listed in Table

2 were calculated. The statistical analysis of each calculated QC metric as well as outliers were visualized in twelve boxplots (Supplementary Figure 1).

To further support the assessment of the participating laboratories´ sequencing quality, the MLST of each isolate was predicted using a command line version of the CGE MLST v 2.0.4 and newest database version available (March 10th, 2021) (Larsen et al., 2012) in addition to a phylogenetic analysis. In specific, single nucleotide polymorphism (SNP) matrices were created for each of the six organisms by using the raw reads of both the culture and corresponding purified DNA submitted by the laboratories. SNPs were determined using the CGE pipeline CSI phylogeny v.1.4 available at (https://cge.cbs.dtu.dk/services/CSIPhylogeny/) (Kaas et al., 2014). The reads were mapped to the reference genomes; using BWA version 0.7.13 (Li, 2013), producing a matrix of SNP differences.

Correlation statistics

The metrics "N50" and "contigs with more than 200 bp" have previously been regarded as the most suitable for estimating sequencing quality (Earl et al., 2011). Here, a linear regression analysis was performed with the purpose of elucidating whether these correlated with other quality metrics and identify those best associated with sequencing quality. Initially, data was cleaned in two steps to ensure trustworthy results, by removing samples larger than 105% or smaller than 95% of assembly size compared to the closed reference genomes or with incorrect or incomplete MLST-scheme genes identified.

Subsequently, data was cleaned for outliers by removing isolates which deviated by more than 3SD from the mean for each sample type. Outlier detection was based on the following metrics: average insert size, quality score of the forward and reverse read separately, total number of contigs, number of contigs more than 200 bp, proportion of reads mapped to the reference genome, N50 and NG50. This restriction was applied as these metrics describe overall quality, while excluded metrics were tied to the yield of the sequencing. Following the exclusion of poor quality data, a linear regression analysis was performed including all the QC parameters found in table 2, using the linear model (lm) in R. Accepted correlation had a p-value less than 0.05 for half (5 out of 10) sample types and Pearson correlations were calculated using the in-built R function (cor). The correlations were visualized using the R-libraries reshape2 and ggplot2 (figure 1).

The SD and mean were recalculated for the cleaned dataset, and a threshold of 3SD below/above the mean, depending on the specific QC-metric (referred to as adjusted quality threshold below) was set to determine laboratories who performed considerably less well comparable to other participants and to define minimum QC-thresholds for acceptable sequence quality. The minimal QC-thresholds for acceptable sequence data was suggested by taking the average of the obtained 3SD levels for each species separately, calculated from the filtered data and excluding laboratories identified as general underperformers.

General underperformance

To identify the laboratories that underperformed overall and need to assess their workflow, a subset of the QC metrics was selected based on the correlation statistics. Values below the adjusted quality threshold in one QC metric, would increase the risk of failing those correlated to it. If a participant was below the adjusted quality threshold across uncorrelated QC metrics, the laboratory is more likely to suffer from issues in their workflow. Failure across these uncorrelated metrics was termed general laboratory underperformance.

Reference

Earl, D., Bradnam, K., st. John, J., Darling, A., Lin, D., Fass, J., Yu, H. O. K., Buffalo, V., Zerbino, D. R., Diekhans, M., Nguyen, N., Ariyaratne, P. N., Sung, W. K., Ning, Z., Haimel, M., Simpson, J. T., Fonseca, N. A., Birol, I., Docking, T. R., … Paten, B. (2011). Assemblathon 1: A competitive assessment of de novo short read assembly methods. *Genome Research*, *21*(12), 2224–2241. https://doi.org/10.1101/gr.126599.111

Kaas, R. S., Leekitcharoenphon, P., Aarestrup, F. M., & Lund, O. (2014). Solving the Problem of Comparing Whole Bacterial Genomes across Different Sequencing Platforms. *Plos One*, *9*(8). https://doi.org/10.1371/journal.pone.0104984

Larsen, M. V., Cosentino, S., Rasmussen, S., Rundsten, C. F., Hasman, H., Marvig, R. L., Jelsbak, L., Sicheritz-Pontén, T., Ussery, D. W., Aarestrup, F. M., & Lund, O. (2012). Multilocus Sequence Typing of Total-Genome-Sequenced Bacteria. *Journal of Clinical Microbiology*, *50*(4), 1355–1361. https://doi.org/10.1128/JCM.06094-11

Li, H. (2013). Aligning sequence reads, clone sequences and assembly contigs with BWA-MEM. *ArXiv E-Prints*, arXiv:1303.3997. https://doi.org/10.48550/arXiv.1303.3997

Li, H., Handsaker, B., Wysoker, A., Fennell, T., Ruan, J., Homer, N., Marth, G., Abecasis, G., & Durbin, R. (2009). The Sequence Alignment/Map format and SAMtools. *Bioinformatics*, *25*(16), 2078–2079. https://doi.org/10.1093/bioinformatics/btp352

Prjibelski, A., Antipov, D., Meleshko, D., Lapidus, A., & Korobeynikov, A. (2020). Using SPAdes De Novo Assembler. *Current Protocols in Bioinformatics*, *70*(1), e102. https://doi.org/https://doi.org/10.1002/cpbi.102
